# Supplementary material for: The Lupus Damage Index Revision Program: Results From the Item Generation and Reduction Phases
Source: Arthritis Care Res (Hoboken). 2026 Aug 12;78(10):1449–66. doi: 10.1002/acr.80102 (PMC13613247; doi:10.1002/acr.80102)
Supplement: Supplementary file 2 [file ACR-78-1449-s002.docx]

**Supplementary Material**

**Table of contents**

**Supplementary Table 1.** List of participants during item generation and item reduction phases of revised SDI development

**Supplementary Table 2.** Candidate items for the revised SDI from literature review and the first round Delphi exercise by organ system

**Supplementary Table 3.** Item reduction decisions by the Item Reduction Committee (n = 190 items)

**Supplementary Table 4.** Excluded items and exclusion reasons by the Clinical Domain Groups

# Supplementary Table 1. List of participants during item generation and item reduction phases of revised Lupus Damage Index development (alphabetically ordered list by surnames)

**SDI Revision Leadership Team**

Ian N. Bruce, Megan R.W. Barber, Ann E. Clarke, Sindhu R. Johnson, Burak Kundakci

**SDI Revision Literature Review Groups**

Jiacai Cho, Nathalie Costedoat-Chalumeau, Hermine Brunner, Megan R.W. Barber, Ellen M. Ginzler, John G. Hanly, Abida Hasan, Colton Hoffer, Murat Inanc, Naureen Kabani, Alexandra Legge, Deborah Levy, Kaitlin Lima, Livia Lindoso, Anselm Mak, Rosalind Ramsey-Goldman, Noelle A. Rolle, Guillermo Ruiz-Irastorza, Clovis A. Silva, Farah Tamirou, Vitor C. Trindade, Évelyne Vinet

**SDI Revision Delphi Panel**

Alfredo Aguirre, Aysun Aksoy, Basma Mohammed Medhat Ali, Gelsomina Alle, Ibrahim Almaghlouth, Khalid A. Alnaqbi, Graciela S. Alarcón, Maame-Boatemaa Amissah-Arthur, Martin Aringer, Laurent Arnaud, Anca Askanase, Sang-Cheol Bae, Sasha Bernatsky, Eloisa Bonfa, Josiane Bourré-Tessier, Ian N. Bruce, Hermine Brunner, Armando A. Calvo Quiroz, Oswaldo Castaneda, François Chasset, Walter Winn Chatham, Kim Cheema, May Y. Choi, Ann E. Clarke, Fabrizio Conti, Nathalie Costedoat-Chalumeau, Adriana Danowski, Alvaro Danza, Dzifa Dey, Hui Jen Ding, Ali Duarte-García, Sergio Duran-Barragan, Doruk Erkan, Jessica English, Serena Fasano, Aurore Fifi-Mah, Cindy Hilary Flower, Paul R. Fortin, Ellen M. Ginzler, Luis Alonso González, Caroline Gordon, Kaien Gu, Gaelle Guettrot-Imbert, Latika Gupta, John Hanly, Julia Harris, Sabrina Hoa, Murat Inanc, Emilia Inoue Sato, Catherine Ivory, Judith James, Sindhu R. Johnson, Andreas Jönsen, Michelle Jung, Diane L. Kamen, Leila Khalili, Amanda King, Marisa Klein-Gitelman, Andrea Knight, Bianca Lang, Aisha Lateef, Juris Lazovskis, Valérie Leclair, Jisoo Lee, Shin-Seok Lee, Alexandra Legge, Dag Leonard, Deborah Levy, S. Sam Lim, Wambui Machua, Meggan Mackay, Anselm Mak, Daniel Martín-Iglesias, Loreto Massardo, Taneisha Khadine Mcghie, Maureen McMahon, Arielle Mendel, Eric Morand, Gabriella Moroni, Marta Mosca, Pintip Ngamjanyaporn, Ola Nived, Sean O'Neill, Pooja Patel, Christine A. Peschken, Michelle Petri, Bernardo Pons-Estel, Guillermo Pons-Estel, Gaafar Mohamed Ragab, Anisur Rahman, Rosalind Ramsey-Goldman, Simona Rednic, John Reynolds, Benjamin Rhodes, Ariadne Katina Riley, Noelle Rolle, Brad Rovin, Guillermo Ruiz-Irastorza, Jorge Sanchez-Guerrero, Syahrul Sazliyana Shaharir, Seung Cheol Shim, Earl Silverman, Christopher Sjöwall, Sarah Skeoch, Frida Strauss, Elisabet Svenungsson, Trudy Taylor, Maria Tektonidou, Julian Thumboo, Homa Timlin, Sofia Tosounidou, Zahi Touma, Manuel F. Ugarte-Gil, Murray Urowitz, Gloria Vasquez, Luis M. Vilá, Évelyne Vinet, Ronald Van Vollenhoven, Alexandre Voskuyl, Anne Voss, Daniel J. Wallace, Joshua Waytz, Victoria P. Werth, Jill Wong

**The SDI Revision Item Reduction Committee**

Laurent Arnaud, Megan R.W. Barber, Ian N. Bruce, Hermine Brunner, Ann E. Clarke, Dafna Gladman, Murat Inanc, Soren Jacobsen, Sindhu R. Johnson, Anselm Mak, Michelle Petri, Rosalind Ramsey-Goldman, Guillermo Ruiz-Irastorza, Alexandre Voskuyl

**SDI Revision Clinical Domain Groups**

Sulaiman Al-Mayouf, Simone Appenzeller, Cynthia Aranow, Bahar Artim-Esen, Anca Askanase, Tadej Avcin, Jo Blair, Hermine Brunner, Laura Bueno, Nathalie Costedoat-Chalumeau, Doruk Erkan, John D. Fisk, Ellen M. Ginzler, John G. Hanly, Colton Hoffer, Murat Inanc, Kenneth Kalunian, Marisa Klein-Gitelman, Alexandra Legge, Deborah Levy, Anselm Mak, Daniel Martín-Iglesias, Joseph F. Merola, Angela Migowa, Takako Miyamae, Gabriella Moroni, Marta Mosca, Gülfer Okumuş, Julie Park, Anisur Rahman, Noelle Rolle, Rosalind Ramsey-Goldman, Brad Rovin, Guillermo Ruiz-Irastorza, Lou Salciccioli, Davinder Singh-Grewal, Eve Smith, Sarah Swauger, Caitlin Tarlton, Frank Sen-Hee Tay, Victoria P. Werth, Samantha Yang, Yew Sen Yuen

# Supplementary Table 2. Candidate items for the revised SDI from literature review and the first round Delphi exercise by organ system

| **Organ system** | **Literature review** | **Both exercises** | **1^st^ round Delphi exercise** |
| --- | --- | --- | --- |
| **Neuropsychiatric** |  | Autonomic disorder; Cognitive impairment; Cranial neuropathy; Demyelinating disorder including Multiple Sclerosis; Ischemic or haemorrhagic stroke; Mood disorder; Peripheral neuropathy; Psychosis; Seizure disorder; Transverse myelitis | Attempted suicide; Cognitive dyslexia; Delirium; Dementia; Encephalitis; Executive function disorder; Hearing impairment; Images changes captured by functional MRI or SPECT; Incapacitating headaches; Insomnia; Loss of sensation; Movement disorders (ataxia, tremors, chorea, hemiballismus); Neurological sequelae (any) from neuropsychiatric SLE; Poor scholastic achievement; Radiographically proven brain atrophy; Tinnitus |
| **Ocular** |  | Acquired enophthalmos; Cataract; Chloroquine and hydroxychloroquine maculopathy; Chronic angle closure, open angle glaucoma and steroid induced glaucoma; Cicatricial conjunctivitis or symblepharon; Cicatricial ectropion (from DLE); Corneal scarring; Madarosis; Optic atrophy; Permanent uniocular or binocular partial or complete loss of vision; Proliferative retinopathy; Retinal necrosis and/or retinal detachment (choroidopathy, lupus retinopathy, serous retinopathy); Scleral thinning (as a result of anterior scleritis) | Keratoconjunctivitis sicca; Optic neuritis; Persistent uveitis or scleritis; Retinal depigmentation and functional loss |
| **Endocrine/**  **Metabolic** |  | Adrenal insufficiency; Anti-insulin receptor antibodies with recurrent hypoglycaemia; Arterial hypertension; Cushing’s Syndrome; Diabetes mellitus; Erectile dysfunction; Hyperlipidaemia; Hypopituitarism; Hypothyroidism; Premature ovarian failure/primary ovarian insufficiency; Reduced ovarian reserve; Testicular dysfunction with reduced sperm count and quality/gonadal dysfunction | Cachexia / Wasting; Chronic fatigue syndrome; Eating disorder; Facial and/or extremity swelling; Hyperthyroidism; Metabolic syndrome; Persistent weight gain; Steroid-induced diabetes |
| **Growth and Development** |  | Delayed puberty; Growth failure; Reduced final height | Non-traumatic long bone fractures for paediatric population |
| **Reproduction/**  **Pregnancy** | Gestational diabetes | Cervical dysplasia; Hypertensive disorders of pregnancy (gestational hypertension, preeclampsia, and/or eclampsia); Infertility; Intra-uterine growth restriction / Small for gestational age; Miscarriage / fetal loss; Placental abruption; Preterm birth; Stillbirth | Congenital heart block; Neonatal lupus in offsprings; Obstetric antiphospholipid syndrome |
| **Hematologic** | Keloid scar linked to lymph node sampling | Functional asplenia; Non-Hodgkin’s lymphoma; Spleen infarction; Splenectomy (for immune thrombocytopenia (ITP)); Splenic atrophy; Treatment-related myelodysplastic syndrome | Antiphospholipid syndrome requiring long-term anticoagulation; Aplastic anaemia; Bone marrow failure (aplasia, myelodysplasia or myelofibrosis); Chronic anaemia; Prolonged cytopenias; Thrombocytopenia, leukopenia |
| **Gastrointestinal** |  | Bowel stricture; Chronic liver disease and cirrhosis including nodular regenerative hyperplasia; Chronic Peritonitis; Dysphagia; Gastroparesis; Malabsorption and Protein Losing Enteropathy; Mesenteric Insufficiency; Motility disorders including oesophageal dysmotility; Pancreatic insufficiency or need to use pancreatic enzyme replacement; Surgical resection of bowel below duodenum, spleen, liver or gallbladder; Upper gastrointestinal tract surgery | Bariatric surgery; Budd-Chiari syndrome; Cholecystitis; Chronic ascites; End stage liver disease; Gastrointestinal bleed; Mesenteric Adenitis; Myositis with dysphagia; Vasculitis involving the mesentery, oesophagus, stomach, peritoneum, gallbladder, pancreas, liver resulting in organ necrosis |
| **Renal** |  | Decreased eGFR; Proteinuria; Renal biopsy findings (Chronicity index >4, OR Interstitial Fibrosis >25%, OR Tubular atrophy >25%) | Biopsy proved renal microangiopathy due to antiphospholipid syndrome even without GFR<50%; Bladder dysfunction; Chronic kidney disease; Chronic sub-nephrotic proteinuria; End-stage renal disease; Haematuria; Haemorrhagic cystitis; Nephritic syndrome; Renal calculi; Renal infarction; Renal tubular defect; Sterile pyuria |
| **Cardiovascular** |  | Accelerated and early atherosclerosis; Angina or coronary artery bypass; Cardiomyopathy (ventricular dysfunction); Complete AV block; Myocardial fibrosis; Myocardial infarction ever; Pericarditis, pericardial thickening or pericardiectomy; QT prolongation; Valve regurgitation; Valve stenosis; Valve thickening; Valve vegetations | Aortitis; Arrythmias; Arterial hypertension; Baseline oxygen requirement; Cardiogenic shock not due to sepsis; Carotid endarterectomy/stenting; Conduction system disease; Diastolic dysfunction; Pulmonary hypertension as a cardiac event; Right ventricular dysfunction |
| **Peripheral vascular** |  | Claudication; Intracardiac thrombus; Minor tissue loss (pulp space); Significant tissue loss ever (e.g. loss of digit or limb); Venous thrombosis or venous stasis | Renal artery stenosis; Severe Raynaud’s; Thrombosis any (arterial or venous) |
| **Pulmonary** | Respiratory insufficiency | Obstructive airway disease; Pleural fibrosis, pleurodesis or pleurectomy for refractory effusions; Pulmonary fibrosis; Pulmonary hypertension; Pulmonary infarction or resection not for malignancy; Shrinking lung | Bronchiectasis; Chronic diffuse alveolar haemorrhage; Diminished lung function (decreased DLCO, FEV1, FVC); Lobectomy; Obstructive Sleep apnoea; Persistent decreased exercise tolerance/DLCO due to ILD or pulmonary emboli; Restrictive lung disease (based on PFT abnormalities) |
| **Musculoskeletal** |  | Atrophy or weakness; Avascular necrosis / Legg-Calve-Perthes disease / Perthes disease; Erosive arthropathy; Fibromyalgia; Fragility fractures; Frailty; Jaccoud’s arthropathy; Muscle atrophy; Osteomalacia; Osteomyelitis; Osteoporosis with fracture; Osteosclerosis; Sarcopenia; Tendon rupture | Chronic gout with erosion and tophi; Inflammatory joint pain; Joint replacement; Osteoporosis even without fracture; Septic arthritis |
| **Skin/**  **Mucocutaneous** | Telangiectasia | Cicatricial skin atrophy; Dyspigmentation (hyperpigmentation or hypopigmentation); Extensive striae; Panniculitis; Periodontal disease with tooth loss; Scarring (cutaneous); Scarring chronic alopecia; Skin ulceration (excluding thrombosis) for > 6 months | Calcinosis; Chronic discoid or subacute lesions; Easy bruisability; Hirsutism; Nasal septum perforation; Onychodystrophy; Persistent malar rash; Post venous thrombosis complications; Severe livedo reticularis; Severe photosensitivity limiting activities of daily living; Sjogren’s syndrome; Xerostomia |
| **Others** |  | Malignancy | Carcinoma in situ (any site); Organ transplantation (non-kidney); Persistent hypogammaglobulinemia; Poverty; Severe or recurrent infections; Social isolation; Treatment related damage; Work disability |

**Supplementary Table 3.** Item reduction decisions by the Item Reduction Committee (n = 190 items)*

*Top-row colors identify individual Item Reduction Committee members. Cell colors denote voting outcomes: blue = include; yellow = possible exclude (e.g. due to overlap); red = strict exclude. Items receiving strict exclude votes from ≥5 Committee members were removed

**Supplementary Table 4.** Excluded items and exclusion reasons by the Clinical Domain Groups

| **Items** | **Exclusion reason** |
| --- | --- |
| **NEUROPSYCHIATRIC** |  |
| Cranial neuropathy | Reversible |
| Movement disorders | Reversible |
| Psychosis | Reversible |
| **GROWTH AND DEVELOPMENT** |  |
| Delayed puberty/Disordered puberty sequences, e.g. reaching menarche without/with minimal/ less than expected growth spurt, breast or pubic hair development | Overlapping, rare |
| **HEMATOLOGIC** |  |
| Splenectomy | Overlapping |
| Bone marrow failure | Reversible |
| Non-Hodgkin’s lymphoma | Overlapping |
| Spleen infarction | Overlapping |
| Treatment-related myelodysplastic syndrome | Reversible, rare |
| **GASTROINTESTINAL** |  |
| Bowel stricture | Rare |
| Chronic liver disease and cirrhosis | Rare |
| Chronic Peritonitis | Rare |
| End stage liver disease | Rare |
| Pancreatic insufficiency or need to use pancreatic enzyme replacement | Rare |
| **PULMONARY** |  |
| Pleural fibrosis, pleurodesis or pleurectomy for refractory effusions | Rare |
| Shrinking lung | Rare |
| **SKIN/MUCOCUTANEOUS** |  |
| Skin ulceration (excluding thrombosis) | Rare |
| Extensive striae | Overlapping |
|  |  |
|  |  |
